# Reconstructing the Islets: Advances in 3D Pancreatic Organoid Models for Functional β-Cell Replacement

**DOI:** 10.3390/ijms27031280

**Published:** 2026-01-27

**Authors:** Muhammad Kamal Hossain, Hyung-Ryong Kim

**Affiliations:** 1 Organoids Laboratory, Department of Pharmacology, College of Dentistry, Jeonbuk National University, Jeonju 54896, Republic of Korea; kamalhossain@jbnu.ac.kr; 2 Non-Clinical Evaluation Center, Biomedical Research Institute, Jeonbuk National University Hospital, Jeonju 54907, Republic of Korea; 3 Department of Pharmacy, Faculty of Pharmaceutical Sciences, University of Science and Technology Chittagong, Chittagong 4202, Bangladesh

**Keywords:** pancreatic organoids, islet architecture, hypoimmunogenic engineering, β-cell maturation, vascularization, immune protection, β-cell replacement

## Abstract

Pancreatic β-cell replacement represents a promising therapeutic avenue for insulin-dependent diabetes, yet clinical translation has been limited by donor scarcity, immune rejection, and incomplete engraftment. Three-dimensional (3D) pancreatic organoids derived from human pluripotent stem cells (hPSCs) or primary tissue offer a scalable and physiologically relevant platform, recapitulating native islet architecture, paracrine interactions, and glucose-responsive insulin secretion. Recent advances in differentiation protocols, vascularization strategies, and immune-protective approaches—including encapsulation and hypoimmunogenic engineering—have enhanced β-cell maturation, survival, and functional performance *in vitro* and *in vivo.* Despite these developments, challenges remain in achieving fully mature β-cells, durable graft function, and scalable, reproducible production that is suitable for clinical use. This review highlights the promise of pancreatic organoid engineering, emphasizing strategies to optimize β-cell maturation, vascular integration, and immune protection, and outlines key future directions to advance organoid-based β-cell replacement toward safe, effective, and personalized diabetes therapies.

## 1. Introduction

Three-dimensional (3D) pancreatic organoids—self-organizing, multicellular structures derived from primary tissue or human pluripotent stem cells (hPSCs/iPSCs)—have emerged as a transformative platform for diabetes research and β-cell replacement therapy [1]. Unlike traditional two-dimensional (2D) β-cell cultures, organoids recapitulate the native islet architecture and multicellular interactions, including paracrine signaling between β, α, δ, and PP cells, as well as interactions with stromal and vascular elements [2]. These features are critical for glucose-stimulated insulin secretion (GSIS), pharmacological testing, and transplantation outcomes. Advances in differentiation protocols that emulate embryonic pancreatic development, coupled with innovations in micro-aggregation and suspension bioreactor culture, have enabled the scalable production of β-like cells and islet-like organoids with improved functional maturation [2,3,4].

Despite these advances, several technical and translational challenges remain. Achieving adult-like β-cell maturity, characterized by dynamic GSIS, metabolic coupling, and stable identity, is still a major bottleneck. Vascularization and oxygenation within organoids are often insufficient, resulting in hypoxia and functional decline post-transplantation [5,6]. Immune rejection presents an additional barrier, necessitating strategies such as encapsulation or hypoimmunogenic genetic engineering to confer durable protection [7,8]. Moreover, scaling production while maintaining reproducibility, quality control, and functional consistency is essential for clinical translation. Integrating tissue engineering, biomaterials, and immune modulation with advanced organoid platforms represents a promising path toward developing functional, transplantable β-cell constructs for diabetes therapy [2,9,10].

With the rapid evolution of organoid biology and the growing success of stem cell-derived β-cell transplantation in early clinical studies, a timely and comprehensive overview of this advancing field is warranted. Progress in pancreatic organoid engineering is transforming our ability to reconstruct islet architecture, investigate diabetes pathophysiology, and develop regenerative strategies for functional β-cell replacement. A deeper understanding of the molecular, metabolic, and structural factors governing β-cell maturation, along with insights into vascular and microenvironmental influences and novel immune-protective approaches, is essential to bridge experimental advances with clinical translation. Moreover, addressing existing challenges related to scalability, reproducibility, and quality control will be crucial for moving toward reliable and clinically applicable organoid systems. By integrating recent advances in differentiation, maturation, vascularization, immune modulation, and translational research, this review outlines current limitations, highlights emerging innovations, and provides a framework for the collaborative efforts needed to achieve fully functional and transplantable β-cell constructs. Overcoming these barriers will accelerate the realization of next-generation regenerative therapies capable of restoring durable glucose homeostasis in patients with diabetes.

## 2. Rationale and Clinical Need of Pancreatic Organoids

Type 1 diabetes (T1D) accounts for approximately 7–12% of all diabetes cases, and the global diabetic population is projected to reach around 700 million by 2045 [11]. T1D is an autoimmune disorder marked by the progressive loss and dysfunction of insulin-producing β-cells. Its pathophysiology involves intricate interactions between damaged or stressed β-cells and immune components—particularly macrophages and T lymphocytes—that trigger pancreatic inflammation and ultimately drive β-cell destruction through apoptotic processes. However, both T1D and advanced insulin-dependent type 2 diabetes are characterized by the loss or dysfunction of pancreatic β-cells [12,13,14], leading to chronic hyperglycemia and severe metabolic complications. Current therapeutic strategies, including exogenous insulin administration, can manage blood glucose but fail to fully recapitulate the dynamic, glucose-responsive insulin secretion of native islets [15,16]. Whole-islet transplantation offers a more physiological approach, yet it is limited by donor scarcity, immune rejection, and progressive graft failure, preventing widespread clinical adoption. To overcome these limitations, recent work increasingly categorizes pancreatic islet reconstruction strategies into certain complementary model systems such as *in vitro*, *in vivo*, pre-clinical, and clinical. In vitro models, including hPSC-derived pancreatic organoids, stem cell-derived islet-like clusters, and microfluidic islet-on-chip platforms provide renewable, highly controlled systems for studying β-cell differentiation, immune interactions, and drug responses while enabling reconstruction of early islet architecture [17]. *In vivo* models using bioengineered scaffolds, vascularized biomaterials, or encapsulated constructs in rodents allow evaluation of graft survival, vascular integration, oxygenation, and glucose responsiveness in a physiological setting [6,18]. Preclinical large-animal models, such as porcine and non-human primate xenotransplantation, supply essential translational data on scale-up, immune compatibility, and long-term graft performance. Clinical models now include stem cell-derived islet replacement therapies under active evaluation [19], where hPSC derived progenitors and fully differentiated islet cells have shown C-peptide resto-ration, improved glycemic control, and partial insulin independence. Collectively, these tiered model systems reinforce the rationale for advancing pancreatic organoid technologies by enabling stepwise refinement from mechanistic *in vitro* studies to clinically relevant validation. For example, in the ViaCyte clinical studies, grafts retrieved after several months *in vivo* exhibited marked patient-to-patient variability. This heterogeneity likely reflects the fact that transplanted cells were delivered at the pancreatic progenitor stage and were required to complete their maturation within the host. In general, β cells would be fully differentiated *in vitro* prior to implantation and possess robust glucose-stimulated insulin-secretion capacity. Transplanting such terminally matured cells would provide a more uniform therapeutic product and could promote faster and more reliable restoration of glycemic regulation [20].

**Figure 1:**
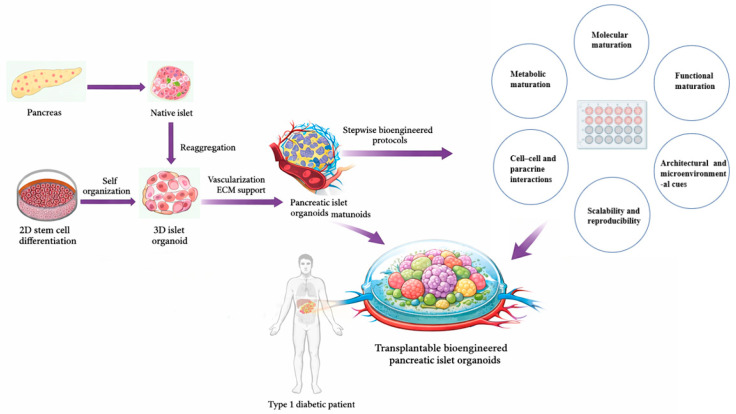
**Stepwise reconstruction of pancreatic islet architecture and functional maturation through 3D organoid engineering.** Schematic overview illustrating the developmental and engineering roadmap toward functional pancreatic islet organoids for β-cell replacement therapy. Native pancreatic islets, derived from the pancreas, serve as the structural and functional blueprint for bioengineering efforts. Stem cells undergo 2D differentiation followed by self-organization into 3D islet organoids, which are further enhanced by vascularization and extracellular matrix (ECM) support. Through stepwise bioengineered protocols, pancreatic islet organoids achieve higher-order molecular, metabolic, and functional maturation, supported by cell–cell and paracrine interactions, architectural cues, and scalability and reproducibility. The final stage involves engraftment of transplantable bioengineered pancreatic islet organoids into Type 1 diabetic patients, aiming to restore physiological insulin regulation.

hPSC-derived pancreatic organoids present a promising solution, offering the potential for scalable production of glucose-responsive β-cells and the ability to engineer constructs that mimic native islet architecture and microenvironment. By reconstructing the cellular composition, paracrine interactions, and vascular niches of native islets, organoid models aim to overcome key barriers of conventional transplantation [21,22,23]. This approach not only holds promise for restoring durable β-cell function in diabetic patients but also provides a versatile platform for disease modeling, drug testing, and the development of personalized regenerative therapies. The conceptual advances in islets organoids align with broader developments in organoid engineering across multiple organ systems. For example, intestinal organoid platforms have adopted analogous strategies for tissue maturation, vascularization, and functional integration [24], underscoring the translational convergence of organoid technologies. An illustrative overview of the progressive strategies for engineering functional pancreatic islet organoids is presented in Figure 1, highlighting the transition from stem cell differentiation to the development and engraftment of transplantable, bioengineered islet constructs for β-cell replacement therapy.

Pancreatic organoids, generated from human pluripotent stem cells or primary pancreatic tissue, are self-organizing multicellular systems that recapitulate the intricate cellular composition and spatial organization of native islets [2]—encompassing endocrine, stromal, and vascular components—thereby enabling coordinated hormone secretion and physiological glucose responsiveness. Guided differentiation protocols that recapitulate embryonic pancreatic development—progressing from definitive endoderm to pancreatic progenitors, endocrine progenitors, and finally β-like cells [25,26,27,28]—have been optimized for 3D culture systems. Advanced suspension cultures and micro-aggregation formats enhance cell–cell and cell–matrix interactions, while stage-specific modulation of signaling pathways such as wingless-related integration site (WNT), transforming growth factor beta (TGF-β), Notch and metabolic conditioning improve β-cell specification and functionality [25,29,30,31]. These platforms enable scalable production of islet-like organoids suitable for preclinical testing, providing a renewable and standardized source of functional β-cells for transplantation, disease modeling, and drug discovery.

## 3. Cellular Origins and Translational Implications of Pancreatic Organoids

Pancreatic organoids can be generated from distinct cellular sources, and the choice of cell-of-origin has direct translational implications for β-cell replacement strategies. Human pluripotent stem cells (hPSCs), including human embryonic stem cell (hESC) and induced pluripotent stem cells (iPSCs), offer an essentially unlimited and standardized cell supply, enabling scalable production of pancreatic progenitors and endocrine cells through developmentally informed differentiation protocols, thereby positioning hPSC-derived organoids as leading candidates for clinically deployable β-cell manufacturing. A key translational advance in hPSC-derived β-cell generation was provided by Hogrebe et al., who demonstrated that cytoskeletal state acts as a decisive regulator of pancreatic lineage commitment. By linking actin polymerization dynamics to endocrine transcriptional programs, the authors showed that excessive cytoskeletal tension suppresses Neurogenin 3 (NGN3)-driven endocrine differentiation, whereas transient actin depolymerization during endocrine induction markedly enhances β-cell maturation and function. Importantly, this cytoskeleton-guided differentiation strategy yielded hPSC-derived β cells exhibiting robust biphasic glucose-stimulated insulin secretion and long-term diabetes reversal *in vivo*, at efficiencies approaching those of primary human islets. These findings underscore cytoskeletal modulation as a critical, and often underappreciated, design parameter for optimizing hPSC-derived pancreatic organoids and advancing their translational readiness for β-cell replacement therapy [32]. In contrast, adult pancreatic ductal cell-derived organoids exhibit robust epithelial self-organization and long-term expandability while retaining pancreatic regional identity, making them attractive platforms for in situ endocrine reprogramming and regenerative medicine approaches rather than direct cell replacement. Seminal studies by Jin et al. and Huch et al. established that the adult pancreas contains expandable progenitor-like epithelial cells capable of long-term organoid growth under defined extracellular matrix and niche signaling conditions [33,34]. Jin et al. demonstrated that colony-forming cells from the adult mouse pancreas can be expanded in Matrigel and subsequently directed toward endocrine or acinar fates in laminin-based hydrogels [33], highlighting the instructive role of matrix context in lineage specification. Complementarily, Huch et al. identified Lgr5^+^ bipotent pancreatic progenitors whose unlimited *in vitro* expansion is sustained by R-spondin-mediated Wnt signaling [34], providing a mechanistic foundation for adult pancreas-derived organoid platforms. Together, these studies underpin adult pancreatic organoids as expandable, lineage-competent systems with translational potential for regenerative and β-cell replacement strategies, although with current limitations in achieving fully mature human β-cell function. In addition, organoids derived from endocrine progenitors or islet-derived cells preserve endocrine lineage memory and exhibit superior functional maturation, glucose-stimulated insulin secretion, and electrophysiological competence [35], but their limited proliferative capacity and donor dependence currently constrain large-scale therapeutic application. These source-specific differences govern organoid self-organization, lineage commitment, and functional maturation, underscoring that rational selection of the starting cell population is a critical design parameter for advancing pancreatic organoids from experimental models toward clinically viable β-cell replacement therapies.

In addition to hPSC- and duct-derived sources, donor variability, disease state, and genetic background are increasingly recognized as critical determinants of islet organoid differentiation efficiency, maturation, and functional stability. Single-cell transcriptomic studies of human islets reveal substantial inter-donor heterogeneity and disease-associated β-cell states that influence insulin secretory capacity and stress resilience [36,37,38,39]. Consistently, stem cell differentiation efficiency and β-cell functional maturation vary across genetic backgrounds [40,41,42]. Moreover, patient-derived pancreatic organoids carrying diabetes-associated mutations exhibit genotype-specific defects in β-cell identity and function [43,44], underscoring the importance of incorporating genetically diverse and disease-relevant donor sources to improve translational relevance and personalized therapeutic development.

## 4. Maturation: A Central Technical Challenge

hPSC-derived β-cells often exhibit fetal-like transcriptional and functional characteristics [45], including immature insulin granules. Achieving full maturation is essential for their therapeutic utility in disease modeling or transplantation, as functional competence—not merely marker expression—determines efficacy. Mature β-cells display robust biphasic GSIS, efficient metabolic coupling, and stable cellular identity [46,47,48], whereas immature cells frequently show poor glucose responsiveness, polyhormonal expression such as insulin–glucagon co-expression, and loss of identity during extended culture or after transplantation [49,50]. These deficiencies impair performance *in vivo*, reflecting the complex microenvironmental requirements of native islets. To address these challenges, several strategies have been employed. Extended *in vitro* culture under optimized nutrient and oxygen conditions, combined with stage-specific small-molecule cues, can enhance metabolic and functional maturation. Co-culture with endothelial and mesenchymal cells, including vascularized organoids or stromal components, provides critical niche signals that support β-cell identity and functionality. In addition, mesenchymal stromal cells have been shown to secrete R-spondin factors that significantly support the proliferation and growth of human pancreatic organoids, highlighting how niche-derived signaling influences organoid expansion and maintenance *in vitro* [51]. *In vivo* engraftment allows organoids to complete maturation by leveraging host physiological cues. Studies have shown that vascularized assembloids and improved oxygenation reduce the loss of β-cell identity and enhance insulin secretion post-transplantation, although hypoxia remains a major cause of functional decline, underscoring the need for advanced vascularization strategies. For example, in the native pancreas, oxygen levels are tightly regulated, maintaining a tension of about 30–40 mmHg to support islet function [52], which relies on one of the body’s richest blood supplies. During isolation and transplantation, these vessels are severed, producing steep gradients where the islet core becomes severely hypoxic. Approximately 70% of transplanted islets experience oxygen tensions near 5 mmHg—substantially lower than the physiological ~40 mmHg [53,54,55], until revascularization begins between days 7 and 14 [56].

Although consensus criteria for defining fully mature β-cells are still evolving, recent studies converge on several widely adopted functional and molecular benchmarks. Mature β-cells generally exhibit a much larger increase in insulin secretion in GSIS assays compared to immature β-cells—for example, neonatal mouse islets showed a ~2.6-fold increase, while more mature islets exhibited ~60-fold increase between low and high glucose [57]. This dramatic difference reflects the acquisition of mature glucose-sensing and secretion coupling mechanisms during postnatal β-cell maturation [58]. Additional functional indicators include the presence of biphasic insulin secretion, synchronized glucose-evoked calcium influx, and improved insulin secretion normalized to DNA or protein content, acknowledging that absolute secretion rates vary across platforms.

At the molecular level, β-cell maturation is supported by increased expression of key transcription factors such as MafA transcription factor (MafA), along with markers including Urocortin 3 (UCN3), and insulin-processing enzymes such as proprotein convertase subtilisin/kexin type 1/2 (PCSK1, PCSK2), and carboxypeptidase E (CPE). Although UCN3 expression can vary depending on species and model systems, these molecular and metabolic indicators collectively provide a practical, though not yet standardized, framework for assessing functional maturation in stem cell-derived β-cell organoids [59,60,61,62,63,64]. Functionally, mature β-cells respond dynamically to physiological glucose through K-ATP channel-mediated depolarization, Ca^2+^ influx, and regulated insulin exocytosis [65]. Metabolically, they shift from glycolysis-dominant activity to oxidative phosphorylation, supporting ATP production and stimulus–secretion coupling [66]. Structurally, proper islet-like clustering, vascularization, innervation, and ECM interactions stabilize β-cell identity and function [67,68,69].

Despite progress, achieving this level of maturation *in vitro* remains a major technical challenge. Current differentiation protocols often yield β-like cells that express relevant markers but remain functionally immature, with weak GSIS and poor insulin kinetics. Limitations include incomplete replication of *in vivo* developmental cues, metabolic immaturity, suboptimal ECM and vascularization, inadequate perfusion, and mechanical constraints within organoid structures. Heterogeneity and dedifferentiation, along with difficulties in scaling production while maintaining reproducibility, further complicate translation. Collectively, these factors highlight that β-cell maturation is both a biological milestone and a technical frontier, critical for advancing functional β-cell replacement and organoid-based diabetes models. Table 1 summarizes these technical challenges and strategies for the maturation of pancreatic organoid models toward functional β-cell replacement.

Accumulating evidence indicates that coordinated 3D cell–cell interactions, dynamic extracellular matrix composition, biomechanical forces, and vascular-associated cues collectively shape endocrine identity and drive the acquisition of glucose-responsive, adult-like β-cell functionality [86]. Organoid systems uniquely recapitulate these multi-scale microenvironmental signals, enabling progressive metabolic maturation, enhanced insulin secretory dynamics, and electrophysiological competence that remain difficult to achieve in conventional two-dimensional cultures. Accordingly, studies employing engineered pancreatic organoids have demonstrated that β-cell maturation is critically dependent on tissue architecture and microenvironmental regulation, underscoring the necessity of organoid-based platforms for generating functionally relevant β cells for translational applications. By extension, precise control of extracellular matrix mechanics and ligand presentation within pancreatic organoids is expected to influence β-cell maturation through integrin-mediated signaling, cytoskeletal tension, and metabolic coupling—processes that are increasingly recognized as determinants of glucose responsiveness and insulin secretory dynamics. For example, a microenvironment-inspired synthetic three-dimensional culture system was developed to decouple and systematically control key biophysical and biochemical niche parameters governing pancreatic organoid behavior, including matrix stiffness, ligand presentation, and degradability. Using pancreatic ductal adenocarcinoma organoids as a model, this study demonstrated that defined synthetic matrices more faithfully recapitulate *in vivo* tissue architecture and signaling states than conventional Matrigel, revealing that organoid phenotype and functional state are highly sensitive to microenvironmental cues [87]. Importantly, although derived from cancer-focused studies, these findings establish generalizable principles of pancreatic organoid maturation and provide a mechanistic foundation for applying engineered, microenvironment–mimetic matrices to guide the progressive maturation and functional competence of non-malignant and stem cell-derived pancreatic organoids. Advancing pancreatic organoid platforms that precisely recapitulate key maturation parameters including coordinated molecular, metabolic, architectural, and microenvironmental cues, will be critical for the reliable generation of functionally stable, glucose-responsive β cells suitable for preclinical testing, disease modeling, and ultimately therapeutic cell replacement.

## 5. Vascularization and Biomaterials

In native pancreatic islets, the vascular network is highly specialized and dense: although islets constitute only ~1% of pancreatic mass, they receive roughly 15% of pancreatic blood flow [88]. Endothelial cells (ECs) within this microvasculature are fenestrated and closely juxtaposed with β-cells, forming intimate interactions with the basement membrane and ECM of the endocrine compartment. This vascular niche supports multiple critical functions, including oxygen and nutrient delivery, metabolic waste clearance, rapid insulin dissemination, endothelial-to-β-cell paracrine signaling, and mechanotransducive cues that maintain β-cell identity and function. The absence or inadequate integration of such a vascular microenvironment represents a major limitation for constructing functional organoids or pseudo-islets for disease modeling or β-cell replacement. More recent studies have begun to address critical microenvironmental limitations in pancreatic organoid platforms. For example, integration of engineered vasculature into pluripotent stem cell-derived islet organoids markedly enhances β-cell functional maturation, demonstrating greater glucose-stimulated insulin secretion and closer recapitulation of *in vivo* islet physiology than non-vascularized counterparts, underscoring the importance of vascular cues in organoid design for diabetes modeling and therapy [89].

Replicating this microvasculature–ECM interface *in vitro* requires careful biomaterial design. Scaffold properties—including matrix composition, adhesive ligand presentation, stiffness, porosity, 3D architecture, and flow dynamics—are essential for organoid survival, endocrine maturation, and functional performance. Despite progress in generating hPSC-derived pancreatic organoids, these constructs often remain embryonic or fetal in function, largely due to missing vascular and ECM-derived cues. Engineering solutions to address these gaps include pre-vascularized organoids via co-culture with endothelial progenitors, incorporation of pro-angiogenic biomaterials, and microfluidic “islets-on-chip” systems that replicate perfusion and nutrient gradients [90,91]. Encapsulation strategies, using materials such as alginate derivatives or hydrogel scaffolds, can simultaneously protect β-cells from immune attack while supporting vascular ingrowth and diffusion at the graft interface. Preclinical studies indicate that pre-vascularized or oxygen-supplemented constructs significantly enhance graft survival and function [92,93,94].

Recent comparative assessments highlight that different vascularization strategies offer distinct strengths and constraints. Endothelial co-culture improves physiological ECM remodeling and supports early vessel development [59], yet often produces heterogeneous vascular density. Microfluidic perfusion systems facilitate controlled shear stress and enhanced oxygenation but require specialized fabrication and technical expertise. Microfluidic systems provide precise control over biochemical gradients, perfusion dynamics, and immune cell interactions, enabling mechanistic interrogation and functional testing under physiologically relevant flow conditions [95]. Integrating organoid constructs into microfluidic or chip-based platforms therefore offers a powerful hybrid strategy to enhance maturation, assess immunocompatibility, and optimize graft performance prior to *in vivo* implantation, rather than representing alternative or competing therapeutic formats. Vascular endothelial growth factor (VEGF)-driven angiogenic induction promotes rapid vascular sprouting; however, excess VEGF can generate aberrant or non-functional vasculature [96]. Three-dimensional bioprinting of pre-patterned vascular channels enables reproducible architecture and superior perfusion capacity, though current bioprinting workflows remain technically demanding and resource-intensive. These complementary strategies underscore that no single method fully recapitulates native islet perfusion, and integrative or hybrid approaches may be necessary to achieve stable and functional vasculature *in vitro*.

Vascularization is indispensable for maintaining islet viability and β-cell functionality. *In vivo*, each β-cell typically resides adjacent to a capillary, ensuring efficient oxygen and nutrient delivery. Beyond perfusion, endothelial cells release bioactive factors, including bone morphogenetic protein (BMP), VEGF, and angiopoietins [97], which regulate β-cell development, survival, and insulin secretion. Disruption of the islet microvasculature during isolation leads to hypoxia, ischemia, and poor graft performance [98], a limitation mirrored in organoid models where diffusion constraints in clusters larger than ~200 μm cause central hypoxia and functional decline [99]. Vascularized pancreatic organoids have demonstrated improved insulin secretion, enhanced β-cell maturity, and superior engraftment outcomes. Recent reports integrating endothelial cells and fibroblasts under perfused conditions have shown that β-cells exhibit more mature phenotypes [100] and stronger calcium-influx responses [101], highlighting the functional importance of endothelial–β-cell crosstalk mediated by BMP2/4-BMPR2 signaling. Co-culture of endothelial and stromal cells, pre-vascularization strategies prior to transplantation, and perfusable microfluidic systems represent key engineering approaches to recapitulate physiological microcirculation.

Additionally, ECM–mimetic scaffolds and basement membrane-inspired hydrogels such as collagen IV, laminin-511, nidogen-based, or functionalized gelatin gels such as PhenoDrive-Y promote EC network formation and β-cell clustering [80], thereby enhancing endocrine functionality. Emerging biomaterial designs also incorporate angiogenic factors such as VEGF, fibroblast growth factor 2 (FGF-2), heparin conjugation, or microchannel architectures that promote vessel ingrowth and perfusion [102]. Advanced bioprinting techniques using pancreas-specific ECM-derived bioinks [103,104] now enable spatially patterned vascular networks within engineered constructs, further bridging the gap between *in vitro* organoids and native islets. Key parameters such as scaffold stiffness, porosity, and interconnectivity must be carefully tuned to balance mechanical support with efficient nutrient and oxygen transport, while remaining compatible with immune-shielding strategies required for transplantation. In line with these design principles, recent optimization of extracellular matrix composition, using biomimetic hydrogels enriched with niche components such as collagen VI, has been shown to enhance the viability, endocrine differentiation, and functional competence of iPSC-derived islet organoids [105], thereby offering a promising approach to improve engraftment efficiency and therapeutic performance. Collectively, vascularization and biomimetic scaffold design stand as central determinants of success for achieving functional, transplantable β-cell organoids that can faithfully model or replace native islet tissue.

## 6. Immune Protection: Encapsulation vs. Hypoimmunogenic Engineering

A major barrier to the clinical translation of β-cell replacement therapies, including pancreatic organoids and stem cell-derived islet-like constructs, lies in the host immune response. Both autoimmune recurrence in T1D and allogeneic rejection can lead to the rapid destruction of transplanted β-cells. Two major strategies have emerged to confer immune protection—physical encapsulation and cell-intrinsic hypoimmunogenic engineering. Each approach offers distinct advantages and limitations in terms of immunological shielding, nutrient exchange, and long-term graft functionality.

### 6.1. Encapsulation Strategies

Encapsulation represents a key immunoprotective approach in β-cell replacement therapy [106], creating a selective physical barrier between transplanted grafts and the host immune system. This strategy prevents direct immune cell infiltration while allowing the diffusion of essential molecules such as oxygen, nutrients, glucose, and insulin. Depending on design and scale, encapsulation can be implemented as either microencapsulation or macroencapsulation. Microencapsulation typically involves coating individual islets or small islet clusters with semi-permeable hydrogels, most commonly based on alginate [107]. Advances in alginate purification and chemical modification—such as triazole–thiomorpholine dioxide derivatization or polyethylene glycol (PEG) functionalization—have significantly improved biocompatibility by minimizing fibrotic overgrowth and enhancing material stability. Macroencapsulation devices, including planar membranes, hollow fibers, and retrievable chambers, permit the transplantation of larger cell masses within a single, retrievable construct. Contemporary systems such as Encaptra^®^ and βAir^®^ integrate oxygenation modules or vascularization-promoting coatings to mitigate diffusion constraints inherent in larger implants [108,109].

Encapsulation strategies preserve graft integrity as an “immune-privileged” entity, thereby reducing or eliminating the need for systemic immunosuppression—an important clinical advantage. Nevertheless, a major challenge lies in achieving an optimal balance between immune isolation and metabolic responsiveness. The semi-permeable materials that provide immune protection also impose diffusion barriers, often resulting in hypoxia, central necrosis, and delayed insulin secretion kinetics. Furthermore, host foreign-body reactions and fibrotic capsule formation around encapsulation devices remain significant impediments to long-term graft survival and function. To address these issues, recent innovations have focused on ultrathin conformal coatings that minimize diffusion distance, immunomodulatory hydrogels incorporating molecules such as C-X-C motif chemokine ligand 12 (CXCL12) or Fas ligand (FasL) to locally suppress immune activation, and vascularization-friendly encapsulation designs that promote host–graft integration without compromising immune isolation [110,111,112]. These emerging biomaterial and engineering advances are refining encapsulation toward more physiologically responsive, durable, and clinically translatable β-cell replacement systems.

### 6.2. Hypoimmunogenic Engineering

An emerging alternative to encapsulation involves the genetic engineering of β-cells or pancreatic organoids to intrinsically evade immune detection, creating so-called hypoimmunogenic or “immune-stealth” grafts. This strategy aims to eliminate the need for physical barriers or systemic immunosuppression by directly modulating cellular immune recognition pathways. At the molecular level, hypoimmunogenic engineering employs targeted genetic modifications to alter the expression of key immune regulatory molecules. Typical approaches include the deletion of β2-microglobulin to ablate major histocompatibility complex (MHC) class I and class II major histocompatibility complex trans activator (CIITA) to silence MHC class II, thereby preventing recognition by cluster of differentiation-positive cells; (CD8^+^ and CD4^+^) T cells, respectively [113]. Additionally, overexpression of immune checkpoint ligands such as programmed death-ligand 1 (PD-L1), CD47, and human leukocyte antigen E (HLA-E) suppresses both T-cell-mediated cytotoxicity and natural killer (NK) cell activation. Further refinements include expression of anti-inflammatory cytokines such as interleukin-10 (IL-10), TGF-β and complement regulatory proteins to promote local immune tolerance within the graft microenvironment.

Recent advances in genome editing technologies, including CRISPR/Cas9 and base editing systems, have enabled precise, multiplexed modification of these immunomodulatory features while preserving β-cell functionality [114]. Hypoimmunogenic β-cells demonstrate the potential for seamless integration with host tissues, avoiding the diffusion and oxygenation limitations associated with encapsulation. Proof-of-concept studies using hPSC-derived hypoimmunogenic β-cells have reported prolonged survival and function in both allogeneic and xenogeneic transplantation models without systemic immunosuppression [115], representing a major milestone toward universal cell therapy products. However, extensive genetic engineering introduces important safety considerations that require critical evaluation before clinical translation. Complete ablation of classical MHC molecules may compromise immune surveillance, increasing susceptibility to viral infection and malignant transformation, as cytotoxic T-cell recognition is a key component of tumor immunosurveillance [116,117,118]. Moreover, loss of MHC class I can paradoxically enhance NK-cell-mediated cytotoxicity through “missing-self” recognition, necessitating compensatory strategies such as HLA-E or CD47 overexpression, which themselves may interfere with immune clearance of aberrant cells [116]. Beyond immunological risks, multiplex genome editing carries potential for off-target mutations, chromosomal rearrangements, and genomic instability, raising concerns about long-term tumorigenicity and functional drift of engineered β-cells, particularly in the context of permanent grafts [114,118]. These safety concerns have prompted a shift toward immune-evasive yet immune-interactive designs that preserve partial antigen presentation or inducible immune recognition, thereby maintaining essential immune communication and surveillance mechanisms while minimizing allogeneic rejection [119]. Therefore, hypoimmunogenic engineering represents a transformative step toward creating universally compatible, safe, and durable β-cell replacement therapies.

These concerns have prompted the translation of immune-evasive engineering principles into organoid-based systems, where immune interactions can be evaluated alongside tissue architecture, lineage specification, and functional maturation. Recent studies have demonstrated that targeted immunoengineering can substantially enhance the survival and function of stem cell-derived islet grafts. Parent et al. showed that selective deletion of human leukocyte antigens in stem cell-derived islets significantly reduced immune recognition and rejection, supporting the feasibility of generating hypoimmunogenic islet cells without compromising endocrine identity or insulin secretory function [120]. Notably, islet-specific organoid studies have shown that immune-evasive engineering can be integrated with pancreatic lineage specification, supporting the feasibility of combining immunoprotection with functional β-cell replacement. Complementing this approach, Yoshihara et al. reported that immune-evasive human islet-like organoids engineered to evade both adaptive and innate immune responses were capable of ameliorating diabetes *in vivo,* highlighting the therapeutic potential of combining organoid architecture with immune-evasion strategies [121]. However, Gerace et al. reported that HLA and PD-L1 modulation alone is insufficient to prevent immune rejection of stem cell-derived islets, whereas engineering β cells to secrete immunoregulatory cytokines such as IL-10, TGF-β, and modified IL-2, promotes regulatory T-cell recruitment and durable immune tolerance [122]. These cytokine-secreting SC-β cells resisted xenograft rejection and restored glycemic control without encapsulation or systemic immunosuppression, providing a strong rationale for incorporating active immune-tolerance strategies into pancreatic organoid-based β-cell replacement approaches. Together, these studies provide compelling evidence that integrating precise immunomodulation with stem cell-derived islet and organoid platforms represents a promising pathway toward durable, transplantation-ready β-cell replacement therapies. Clinical success is dependent not only on immune evasion but also on preserving immune surveillance, genomic integrity, and long-term safety. Rational immune-interactive design principles, combined with rigorous genomic and oncologic monitoring, will therefore be essential for translating immune-evasive islet organoids into durable and clinically acceptable therapies.

### 6.3. Integrating Immune Protection with Organoid Platforms

In the context of pancreatic organoid systems, immune protection strategies must be re-envisioned to accommodate the intricate cellular organization, vascular-like networks, and metabolic demands characteristic of physiologically relevant islet-like tissues. Traditional encapsulation approaches are being reengineered to integrate seamlessly with organoid culture systems, employing biomaterials that support not only immune isolation but also cell–cell communication, oxygenation, and dynamic glucose responsiveness. Advances in hydrogel chemistry and biofabrication have enabled the development of encapsulation matrices tailored to organoid assembly and vascularization [123], thereby maintaining both immune privilege and metabolic efficiency. In parallel, the application of hypoimmunogenic engineering to organoid-derived β-cell progenitors presents a complementary, cell-intrinsic strategy for achieving immune tolerance. By genetically modifying organoid-forming cells to express immune-evasive or immunomodulatory molecules, researchers can create scalable, self-protective tissues that retain the architectural and functional fidelity of native islets. Recent studies suggest that these engineered organoids sustain insulin secretion and cellular viability in allogeneic environments without the need for systemic immunosuppression [121], highlighting their translational promise.

**Figure 2:**
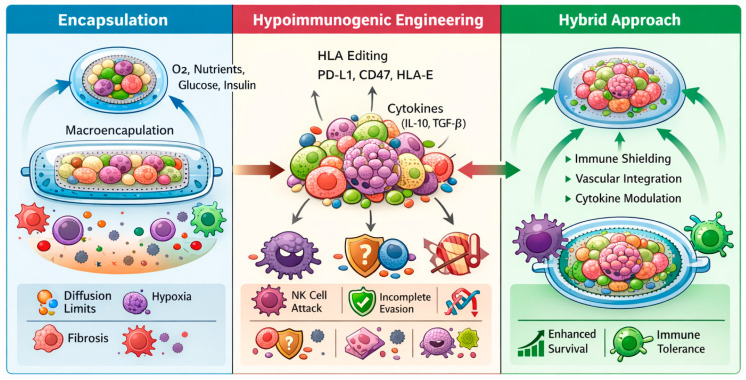
**Immune protection strategies for pancreatic organoid-based β-cell replacement.** This schematic illustrates three complementary immune-protection paradigms applied to pancreatic organoids and stem cell-derived islet-like constructs. (**Left**) Encapsulation strategies, including micro- and macroencapsulation, physically isolate transplanted organoids from host immune cells while permitting diffusion of oxygen, nutrients, glucose, and insulin; key limitations such as diffusion constraints, hypoxia, and fibrotic foreign-body responses are indicated. (**Middle**) Hypoimmunogenic engineering approaches involve genetic modulation of immune recognition pathways, including HLA editing and expression of immune checkpoint molecules (e.g., PD-L1, CD47, HLA-E) and immunoregulatory cytokines (e.g., IL-10, TGF-β), enabling host integration but remaining vulnerable to incomplete immune evasion and natural killer cell-mediated clearance. (**Right**) An emerging hybrid strategy integrates partial encapsulation with targeted immunoengineering to enhance vascular integration, immune tolerance, and long-term graft survival.

A hybrid paradigm is now emerging that integrates both encapsulation and hypoimmunogenic modification to exploit the synergistic advantages of each approach [124]. Encapsulated hypoimmunogenic β-cell organoids exhibit enhanced graft survival, reduced fibrotic encapsulation, and improved metabolic responsiveness compared to either strategy alone. As illustrated in Figure 2, future advances in pancreatic organoid-based β-cell replacement are likely to rely on hybrid immune-protection strategies that combine partial encapsulation with targeted immunoengineering to balance immune tolerance, vascular integration, and long-term safety. Such convergent approaches may overcome the limitations of single-modality strategies and accelerate the development of durable, immunosuppression-free therapies for diabetes.

## 7. Scaling, Reproducibility and Quality Control

One of the major translational challenges in developing clinically relevant pancreatic organoids or islet-like constructs lies in achieving scalable, reproducible, and quality-controlled production. For β-cell replacement therapies, large numbers of functionally mature and glucose-responsive cells are required, while heterogeneity or batch variability can compromise graft performance, safety, and regulatory approval. Scaling up production from small laboratory formats to clinical-scale bioprocesses demands optimization of multiple parameters, including culture format such as 2D versus 3D; adherent versus suspension, aggregate size uniformity, oxygen and nutrient diffusion, and cost-efficient throughput. Recent progress in suspension bioreactors and microfluidic platforms has demonstrated the potential to generate up to 10^4^–10^5^ organoids in a single batch, while systems such as Vertical Wheel^®^ bioreactors have enabled scale-up from 0.1 L to 0.5 L volumes without compromising structural integrity [125,126]. Despite these advances, maintaining consistency across batches and pluripotent stem cell lines remains a key bottleneck due to intrinsic biological variability, differentiation efficiency, and epigenetic drift. Reproducibility across different donors, cell lines, and laboratories requires standardization of protocols, automated handling, and precise environmental control. Emerging organoid-on-a-chip systems are addressing this by providing stable nutrient flow, mechanical cues, and real-time monitoring to reduce stochastic variation during differentiation.

Establishing robust quality control (QC) and release criteria is essential for translating pancreatic organoids into therapeutic products. Core QC parameters include identity, defined by the expression of β-cell markers such as PDX1, NKX6.1, MafA, and insulin; purity and safety, assessed through detection of residual pluripotent cells, genomic stability, and sterility; and potency, validated via GSIS assays and *in vivo* efficacy in diabetic models. Additional QC measures include organoid morphology and size uniformity, oxygen consumption rates, and multi-omics profiling to verify maturity and minimize off-target populations. Process control metrics—such as fixed seeding density, metabolic monitoring, sterility testing, and traceability—ensure compliance and reproducibility. Despite encouraging progress, several challenges persist, including incomplete β-cell maturation, high production costs, mass transfer limitations within large organoids, and batch-to-batch variance due to donor or iPSC line differences. Moreover, the absence of standardized QC benchmarks and harmonized regulatory frameworks complicates clinical translation. Future directions should focus on integrating automated, closed-system bioprocesses with real-time analytics, xeno-free reagents, and defined maturation cues to ensure consistency, scalability, and regulatory-grade quality. Such advances are crucial for establishing pancreatic organoid-based β-cell replacement as a viable, safe, and reproducible therapeutic platform.

## 8. Preclinical and Early Clinical Progress

The translation of pancreatic organoids and stem cell-derived islet-like constructs into viable therapeutic platforms have advanced significantly in both preclinical models and early human clinical trials. In animal studies, hPSCs—including embryonic stem cells (ESCs) and induced pluripotent stem cells (iPSCs)—have been successfully differentiated into pancreatic progenitors or β-like cells, aggregated into organoid or islet-like clusters, and transplanted into diabetic rodents and non-human primates to restore glucose control. Notably, hPSC-derived endocrine clusters transplanted into streptozotocin (STZ)-induced diabetic mice normalized blood glucose within days, demonstrating superior efficacy over 2D differentiated counterparts, largely due to improved cell–cell signaling, paracrine factor retention, and enhanced survival under metabolic stress conditions [127,128]. Incorporating 3D scaffolds or hydrogels, such as Amikagel further improved organoid architecture, islet marker expression (PDX1, NKX6.1), insulin/C-peptide secretion, and functional engraftment *in vivo* by promoting spatial organization, oxygen diffusion, and extracellular matrix-mediated maturation cues [123].

However, several preclinical strategies failed to achieve durable metabolic correction due to rapid post-transplant hypoxia, insufficient vascular integration, inflammatory foreign body responses, and incomplete endocrine maturation, which collectively limited β-cell survival and delayed glucose-responsive insulin kinetics. Efforts incorporating endothelial or stromal co-culture improved early engraftment but often showed declining graft performance over time, highlighting that vascularization alone is insufficient without concurrent immune protection and metabolic maturation. Decellularized scaffolds reduce immune rejection while preserving the natural architecture and biochemical cues of native organs. They are being explored in whole-organ engineering by repopulating these matrices with patient-derived cells, offering a promising strategy to address donor shortages, although this approach is still largely in the preclinical phase [129,130].

Early clinical programs have extended these findings into humans. ViaCyte Inc. pioneered the transplantation of hPSC-derived pancreatic endoderm (PEC-01) cells encapsulated within macro- or micro-encapsulation devices for patients with T1D [131]. Initial trials such as NCT02239354 demonstrated safety without systemic immunosuppression, but engraftment and insulin production were limited due to inadequate vascularization, foreign body fibrotic encapsulation, and diffusion barriers restricting oxygen and nutrient delivery. Subsequent cohorts using semi-open devices combined with immunosuppressive regimens achieved detectable C-peptide levels and modest glycemic improvements [132], indicating that immune isolation alone was insufficient to support functional maturation *in vivo*.

**Figure 3:**
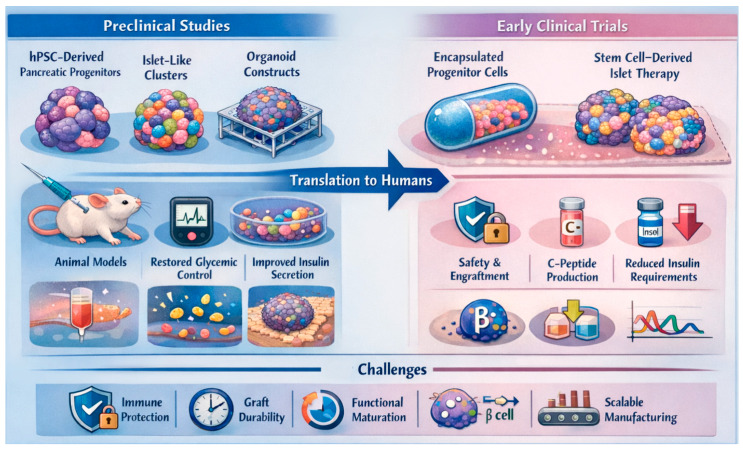
**Preclinical and early clinical translation of pancreatic organoid-based β-cell replacement therapies.** This schematic illustrates the translational pipeline from preclinical development to early human application of stem cell-derived pancreatic progenitors, islet-like clusters, and organoid constructs for diabetes therapy. In animal models, hPSC-derived β-like cells organized into three-dimensional organoids or scaffold-supported platforms restore glycemic control, enhance glucose-stimulated insulin secretion, and improve graft maturation and engraftment compared with conventional two-dimensional cultures. Early clinical programs using encapsulated pancreatic progenitors and fully differentiated stem cell-derived islet products demonstrate safety, engraftment, endogenous C-peptide production, and reductions in exogenous insulin requirements, although outcomes remain variable. Persistent challenges include immune protection, long-term graft durability, functional maturation, and scalable manufacturing for broad clinical translation.

More recently, Vertex Pharmaceuticals reported promising outcomes using a stem cell-derived islet cell therapy (VX-880), where all treated T1D patients showed engraftment and glucose-responsive insulin production within 90 days, and most achieved near-complete insulin independence (11 of 12 patients reduced or eliminated insulin use, and most achieved HbA_1_c < 7.0%) [133,134]. These superior outcomes are attributed to the transplantation of terminally differentiated endocrine cells rather than progenitors, rapid vascular integration under immunosuppression, and improved manufacturing consistency, enabling faster functional maturation and higher effective β-cell mass. Similarly, autologous endoderm stem cell-derived islet tissue transplanted into a patient with type 2 diabetes yielded near-complete insulin independence and excellent glucose time-in-range within weeks [135], underscoring the importance of immunologic compatibility and patient-specific cell sourcing in promoting durable graft survival. Collectively, these trials confirm the feasibility, safety, and functional potential of stem cell-derived and organoid-based β-cell therapies. However, clinical outcomes remain heterogeneous, reflecting variable graft survival, immune-mediated attrition, differences in device performance, and inconsistent endocrine maturation across recipients. Several trials report modest reductions in HbA_1_c, approximately 0.5–1.2% alongside increases in C-peptide output, confirming engraftment and graft-derived insulin production [136,137]; however, these improvements are typically observed in only a subset of participants rather than uniformly across cohorts. Interpretation remains cautious due to small patient numbers, short follow-up intervals; generally, 6–12 months, and heterogeneity in graft survival and endocrine performance, which collectively limit the ability to draw definitive conclusions. Although these initial studies demonstrate compelling proof-of-concept for replacing pancreatic islets with stem cell-derived products, robust evidence from larger, extended-duration, and randomized clinical trials will be required to confirm sustained therapeutic benefit and inform future clinical application. These limitations underscore that early graft loss from hypoxia, delayed vascularization, and immune rejection—rather than differentiation inefficiency alone—remain dominant barriers to sustained clinical efficacy. In this context, early-phase clinical studies are now evaluating fully differentiated stem cell-derived islet organoids with improved maturation states and immune protection strategies. The ongoing Phase 1/2 trial (NCT06415643) is evaluating the safety, tolerability, and early metabolic efficacy of transplanting fully differentiated stem cell-derived islet organoids into individuals with type 1 diabetes. This study also seeks to address key translational barriers—including immune protection, long-term graft performance, and manufacturing scalability—and represents one of the first clinical efforts to bring islet-organoid-based therapy into the human setting [138]. The translational trajectory of organoid-based β-cell replacement therapies is outlined in Figure 3, which integrates preclinical successes with early clinical experience and underscores key remaining barriers, including immune protection and maturation.

For pancreatic organoid models, these findings underscore the importance of bridging *in vitro* functionality with *in vivo* efficacy. Future organoid-based constructs must demonstrate durable engraftment, vascular integration, and dynamic insulin responsiveness comparable to native islets. Integrating immune-evasive genetic engineering with pro-vascular biomaterials and optimizing scale, reproducibility, and quality control will be central to clinical translation. As ongoing clinical trials expand in scope and duration, standardizing dosage, potency assays such as glucose-stimulated insulin secretion kinetics, and GMP-compliant manufacturing will be crucial for regulatory approval. While preclinical and early clinical results remain preliminary, they strongly support the viability of pancreatic organoids as a next-generation platform for β-cell replacement therapy—linking stem cell biology, biomaterials engineering, and immunomodulation into a cohesive translational strategy.

## 9. Remaining Gaps and Research Priorities

Despite substantial progress in pancreatic organoid engineering and stem cell-derived islet-like constructs, several unresolved biological and translational challenges continue to limit their clinical applicability. A central barrier remains the incomplete functional maturation of β-like cells. Most current organoid systems retain immature transcriptional profiles and exhibit blunted or monophasic glucose-stimulated insulin secretion, thereby limiting their capacity to dynamically regulate glycemia *in vivo*. Although co-culture with endothelial or stromal cells, refinement of three-dimensional architecture, and exposure to metabolic or mechanical cues have shown potential to enhance maturation, these approaches remain insufficiently standardized and incompletely optimized. Vascularization and oxygen delivery represent closely related constraints. Large organoid aggregates are particularly susceptible to hypoxia and central necrosis, which compromise β-cell survival and long-term function. While prevascularized scaffolds, endothelial progenitor incorporation, and angiogenic signaling strategies have demonstrated encouraging preclinical results, achieving rapid, stable perfusion without inducing fibrosis or chronic inflammation remains an unmet challenge.

Immune rejection and inflammatory responses constitute another major translational bottleneck. Current protection strategies primarily rely on either physical immune isolation via encapsulation or immune evasion through hypoimmunogenic genome editing. Encapsulation provides immediate immunoprotection but is frequently undermined by diffusion limitations and fibrotic foreign-body responses that progressively impair insulin secretion. Conversely, hypoimmunogenic engineering enables direct host integration and improved functional maturation but raises concerns regarding incomplete immune evasion, natural killer cell-mediated clearance, and long-term genomic and tumorigenic safety. These limitations highlight the need for balanced immunomodulatory approaches rather than reliance on single-strategy solutions.

Notably, many translational failures in β-cell replacement can be attributed to insufficient modeling of human-specific immune interactions, overreliance on short-term metabolic endpoints, and the absence of standardized benchmarks for defining clinically meaningful β-cell maturity and durability. The lack of immune-competent and vascularized pancreatic organoid models further limits the predictive value of preclinical studies. Clinical studies demonstrate that sustained metabolic benefit after islet transplantation requires preservation of a large functional β-cell mass, yet most recipients exhibit progressive graft attrition over time, with residual function often insufficient to maintain insulin independence [139]. Even under optimized metabolic control, endogenous β-cell function continues to decline over several years, underscoring the intrinsic vulnerability of β-cells to long-term survival stresses [140]. Together, these findings support the conclusion that engraftment efficiency and long-term graft viability remain suboptimal, with many grafts retaining only a small fraction of functional β-cells within months of transplantation.

Beyond biological challenges, scalable and reproducible manufacturing remains a critical gap. Clinical translation will require the production of large quantities of organoids with consistent differentiation efficiency, purity, and functional performance. Batch-to-batch variability, donor-dependent heterogeneity, and safety concerns, including residual pluripotent cells and genomic instability, underscore the need for automated bioreactor platforms, standardized potency assays, and long-term quality surveillance aligned with regulatory requirements. Finally, integration with host physiology remains incompletely addressed. Native pancreatic islets operate within complex neuroendocrine, hormonal, and intercellular feedback networks that are only partially recapitulated by current organoid platforms [141,142]. In parallel, patient selection will be a critical determinant of early clinical success for islet organoid-based therapies. Given the procedural risks and uncertainties surrounding immune-modulatory strategies, initial trials are likely to focus on individuals with labile diabetes, hypoglycemia unawareness, or advanced complications, in whom the potential benefits outweigh intervention-associated burdens. However, such targeted deployment may limit population-level impact unless advances in manufacturing scalability, delivery platforms, and cost efficiency enable broader clinical adoption. Moreover, economic feasibility, equitable access, and regulatory harmonization will play central roles in shaping real-world implementation.

Looking ahead, the most promising path forward lies in hybrid strategies that combine partial immune shielding with targeted immunoengineering, alongside the incorporation of vascular, stromal, and immune components to enhance physiological relevance. Over the coming years, key research priorities should be focused on (i) establishing standardized criteria for β-cell functional maturity, glucose responsiveness, and safety; (ii) developing immune-aware and vascularized pancreatic organoid systems; (iii) implementing scalable, reproducible, and regulatory-compliant manufacturing pipelines; and (iv) conducting long-term, longitudinal assessments of graft stability, immune interactions, and metabolic control. Addressing these priorities will be essential for advancing pancreatic organoid technologies from experimental constructs to clinically viable therapies for diabetes.

## 10. Conclusions

Three-dimensional pancreatic organoids represent a transformative platform for β-cell replacement, offering the potential to overcome the longstanding limitations of donor scarcity, immune rejection, and incomplete engraftment in diabetes therapy. By recapitulating key features of native islets—including multicellular architecture, paracrine signaling, and glucose-responsive insulin secretion—organoid systems provide both a scalable source of β cells and a physiologically relevant model for disease research and drug testing. Advances in differentiation protocols, vascularization strategies, and immune-protective approaches have markedly improved the functionality and transplantation ability of these constructs, yet significant challenges remain. In particular, achieving full β-cell maturation, long-term survival post-transplantation, and reproducible large-scale production are critical hurdles that must be addressed before widespread clinical application. Future efforts will require an integrated, multidisciplinary approach that combines stem cell biology, tissue engineering, immunology, and translational medicine. Key directions include the development of fully vascularized and ECM–mimetic organoids that faithfully recapitulate the native islet microenvironment, the incorporation of immune-evasive or encapsulation strategies to ensure graft survival without systemic immunosuppression, and the optimization of scalable, GMP-compliant manufacturing processes with rigorous quality control. Additionally, patient-specific strategies, such as autologous iPSC-derived grafts or universal hypoimmunogenic organoids, hold promise for personalized therapy. In conclusion, the convergence of these approaches has the potential to realize durable, physiologically functional β-cell replacement, transforming the treatment landscape for diabetes and advancing regenerative medicine toward clinically viable solutions.

## Figures and Tables

**Table 1:** Technical challenges and strategies for the maturation of pancreatic organoid models toward functional β-cell replacement.

Aspect	Hallmarks	Challenges in Organoid Models	Strategies to Enhance Maturation	References
**Molecular maturation**	Expression of adult β-cell markers such as MafA ↑, UCN3 ↑, PCSK1/2 ↑, Insulin (INS) ↑, NK6 homeobox 1 (NKX6.1) ↑; reduced fetal markers (MafB ↓).	Incomplete transcriptional switch; persistent immature or polyhormonal phenotypes (Insulin-positive, glucagon-positive: INS^+^GCG^+^).	Stage-specific induction of MafA; thyroid hormone (T3) or estrogen-related receptor gamma (ERRγ) agonists to drive transcriptional maturation.	[46,70,71]
**Metabolic maturation**	Switch from glycolytic to oxidative phosphorylation; increased mitochondrial activity and ATP coupling to KATP channels.	Limited mitochondrial biogenesis and oxidative capacity; glycolytic bias *in vitro*.	Activation of ERRγ; modulation of mammalian target of rapamycin/AMP-activated protein kinase (mTOR/AMPK); lipid supplementation; dynamic glucose exposure mimicking feeding cycles.	[72,73]
**Functional maturation**	Robust GSIS; appropriate calcium flux and insulin granule exocytosis.	Blunted or static GSIS; poor calcium oscillations; incomplete coupling of metabolism to secretion.	Dynamic glucose training; co-culture with α/δ-cells to enable paracrine feedback; extended maturation culture (>4–6 weeks).	[74,75,76,77]
**Architectural and microenvironmental cues**	Three-dimensional islet-like architecture; vascularization; ECM-integrin signaling; oxygen/nutrient gradients akin to native islet.	Organoids lack perfusion, vascular networks, and ECM diversity; hypoxia in large clusters.	Co-culture with endothelial cells; biomaterial scaffolds (Collagen type II; COL2/Collagen type V; COL5, laminin-rich hydrogels); microfluidic perfusion bioreactors.	[78,79,80]
**Cell–cell and paracrine interactions**	Balanced β:α:δ cell ratios; paracrine insulin–glucagon–somatostatin regulation.	Limited heterotypic integration; random spatial distribution of endocrine subtypes.	Guided differentiation toward mixed endocrine populations; organoid assembly using single-cell patterning or organ-on-chip integration.	[40,81]
**Epigenetic and long-term stability**	Stable β-cell identity, reduced dedifferentiation markers, e.g., Regulatory factor X6; (RFX6) ↑, Pancreatic and duodenal homeobox 1 (PDX1) ↑.	β-cell identity loss upon prolonged culture or transplantation; epigenetic instability.	Long-term culture in defined media; small molecules promoting chromatin remodeling (Histone deacetylase; HDAC inhibitors); *in vivo* conditioning post-transplantation.	[82,83,84]
**Scalability and reproducibility**	Consistent generation of mature β-cells across batches and donors.	Variability in differentiation outcomes; immature populations dominate large-scale production.	Automated differentiation platforms; bioreactors with real-time oxygen/nutrient control; synthetic ECM gels with defined stiffness.	[41,85]

## Data Availability

The study is based solely on previously published literature. No datasets were generated or analyzed. All sources of information are cited in the reference list.

## Abbreviations

The following abbreviations are used in this manuscript:

GSIS	Glucose-stimulated insulin secretion
T1D	Type 1 diabetes
WNT	Wingless-related integration site
TGF-β	Transforming growth factor beta
NGN3	Neurogenin 3
MafA	MafA transcription factor
PCSK1	Proprotein convertase subtilisin/kexin type 1
PCSK2	Proprotein convertase subtilisin/kexin type 2
CPE	Carboxypeptidase E
UCN3	Urocortin 3
K-ATP	ATP-sensitive potassium channels
INS	Insulin
NKX6.1	NK6 homeobox 1
INS^+^GCG^+^	Insulin-positive, glucagon-positive
ERRγ	Estrogen-related receptor gamma.
mTOR	Mammalian target of rapamycin
AMPK	AMP-activated protein kinase.
COL2	Collagen type II
COL5	Collagen type V
RFX6	Regulatory factor X6
PDX1	Pancreatic and duodenal homeobox 1
HDAC	Histone deacetylase.
BMP	Bone morphogenetic protein.
VEGF	Vascular endothelial growth factor.
FGF-2	Fibroblast growth factor 2
CXCL12	C-X-C motif chemokine ligand 12
FasL	Fas ligand
CIITA	Class II major histocompatibility complex transactivator
CD4^+^	Cluster of differentiation 4-positive cells
CD8^+^	Cluster of differentiation 8-positive cells
PD-L1	Programmed death-ligand 1
HLA-E	Human Leukocyte Antigen E
IL-10	Interleukin-10
HbA_1_c	Glycosylated hemoglobin
